# Effects of soy consumption on metabolic parameters in patients with metabolic syndrome: A systematic review and meta-analysis

**DOI:** 10.17179/excli2021-3348

**Published:** 2021-03-16

**Authors:** Noushin Mohammadifard, Firoozeh Sajjadi, Fahimeh Haghighatdoost

**Affiliations:** 1 Hypertension Research Center, Cardiovascular Research Institute, Isfahan University of Medical Sciences, Isfahan, Iran; 2 Interventional Cardiology Research Center, Cardiovascular Research Institute, Isfahan University of Medical Sciences, Isfahan, Iran; 3 Isfahan Cardiovascular Research Center, Cardiovascular Research Institute, Isfahan University of Medical Sciences, Isfahan, Iran

**Keywords:** soy, metabolic syndrome, hypertriglyceridema, hyperglycemia, blood pressure, meta-analysis

## Abstract

Functional foods like soy have unique effects on health status. Although various dietary modifications have been recommended to ameliorate features of metabolic syndrome (MetS), no meta-analysis has summarized the effect of soy consumption in patients with MetS. PubMed, ISI Web of Science, and Scopus were searched for relevant articles until Jun 2020, resulting in six relevant publications that were included in our meta-analysis. Combining a total of 9 comparisons suggested a significant decrease in serum levels of triglyceride (WMD: -0.29; 95 % CI: -0.49, -0.09 mg/dL), total cholesterol (WMD: -1.46; 95 % CI: -1.70, -1.22 mg/dL), LDL-C (WMD: -0.73; 95 % CI: -0.93, -0.52 mg/dL) and no change in serum HDL-c levels. Combining a total of 7 effect sizes examining soy effects on glycemic parameters indicated that subjects who consumed soy products had lower levels of fasting blood sugar (WMD: -0.90; 95 % CI: -1.12, -0.68 mg/dL), insulin (WMD: -1.06; 95 % CI: -1.29, -0.84 pmol/ L) and HOMA-IR (WMD: -1.08; 95 % CI: -1.31, -0.85) compared with those in the control group. Soy consumption could not significantly change anthropometric measures and blood pressure. Consuming soy products in patients with MetS effectively improved lipid profile and glycemic parameters independent of affecting anthropometric measures.

## Introduction

Metabolic syndrome (MetS) is a cluster of various metabolic abnormalities including elevated blood pressure, insulin resistance, abdominal obesity and dyslipidemia, which causes a major public health concern (Rochlani et al., 2017[37]). MetS promotes cardiovascular disease (CVD) progression by threefold (O'Neill and O'Driscoll, 2015[31]) which is the leading cause of global death and burden of disease (Stanaway et al., 2018[45]). Although the exact etiology of MetS has not been fully understood, it is proposed that diverse factors consisting of genetic, metabolic and environmental characteristics contribute to the development of MetS and its components (Xu et al., 2018[51]). Dietary modification is the crucial approach for prevention and treatment of MetS (de la Iglesia et al., 2016[11]). Functional foods and nutraceuticals have a main role in the prevention of CVD and its risk factors (Ruscica et al., 2014[39]).

Soy, as a functional food, is known as a cardioprotective food (Alissa and Ferns, 2012[2]) because of its high content of fiber, protein, minerals, B vitamins, phytosterols, polyphenols, isoflavones, antioxidants and unsaturated fatty acids with low amounts of saturated fats (Rizzo and Baroni, 2018[36]). Some meta-analyses and randomized clinical trials (RCTs) indicated beneficial effects of soy on glycemic, inflammatory, thrombotic and oxidative stress responses (Bakhtiari et al., 2019[7]; Mazidi et al., 2016[24]; Rios et al., 2008[35]). However, different types of studies, which varied from laboratory to human studies, including RCTs and large-scale observational studies have provided conflicting findings of the impact of soy on serum lipids, blood pressure, glycemic and obesity status (Bakhtiari et al., 2019[7]; Ruscica et al., 2018[40]; Zhang et al., 2016[54]; Yang et al., 2005[52]). 

It might be explained by various study designs or duration, the type of soy-based foods including soybeans, different soy products or isolated components, diverse target populations and eligibility criteria (Bakhtiari et al., 2019[7]; Ruscica et al., 2018[40]; Zhang et al., 2016[54]). In addition, nutrients' content and bioavailability of different soy products vary by their processing methods and phytate content (Cassidy et al., 2006[9]). 

Several meta-analyses examining the effect of soy products on metabolic parameters have provided beneficial effects of soy on insulin resistance (Liu et al., 2011[19]) and serum lipids (Tokede et al., 2015[48]). Given that MetS is a cluster of metabolic abnormalities, and insulin resistance is the main culprit of the abnormalities in MetS (Reaven, 1995[34]), soy products, possibly depending on their types, may be a useful food choice implicated in the treatment of MetS. Although some clinical trials have evaluated the effect of soy consumptions in patients with MetS, they are inconsistent in their results. Therefore, due to the lack of any systematic review and meta-analysis in this context, we conducted a systematic review of RCTs in an attempt to summarize the evidence on the effect of consuming whole soy, soy products and its isolated components on Mets features in adults. 

## Materials and Methods

### Search strategy and data sources

This meta-analysis was performed according to the Preferred Reporting Items for Systematic Reviews and Meta-Analyses statement (Moher et al., 2009[26]). PubMed, Web of Science, and Scopus were searched from inception up to 1 June, 2020 for published RCTs examining the effect of soy-based foods on metabolic parameters in patients with MetS. Search terms were defined using Medical Subject Headings (MeSH) and included “Soybeans” or “soybean” or “soy” or “soy foods” or “soy food” or “Tofu” or “miso” or “tempeh” or “natto” or “Fabaceae” or “legumes” or “legume” or “ Soy Milk” or “Soybean Proteins” or “Isoflavones” or “daidzein” or “genistein” or “phytoestrogens” or isoflavone* or “soya” in combination with "Metabolic Syndrome" or "MetS" or "syndrome X" or "Insulin Resistance Syndrome X" or "Metabolic X Syndrome" or "Dysmetabolic Syndrome X" or "Reaven Syndrome X" or "Metabolic Cardiovascular Syndrome". In addition, the database search was accompanied with a manual hand search of reference lists of retrieved articles as a complement. No restriction was made on the publication date and articles' language. 

### Study selection

Two independent investigators (NM and FH) screened the titles and abstracts of all retrieved articles in the initial literature search, and disagreements were resolved by consensus. After applying inclusion and exclusion criteria, the full texts of relevant articles were further evaluated.

Articles were included in this meta-analysis if they were RCT, conducted among patients with MetS, aged > 18 years, and they were prescribed any soy-based foods (except for soybean oil), and reported means of any metabolic parameters including serum lipids (TG, TC, LDL-C, and HDL-C), glycemic markers (FBS, insulin, HOMA-IR), systolic and diastolic blood pressure, and anthropometric measures (weight, BMI, and WC) along with their corresponding standard deviation (SD) or standard error (SE) or interquartile range (IQR) or 95 % confidence intervals (CI) before and after intervention. Studies that assessed the effects of supplementary soy in animals, or laboratory researches as well as supplementary with soy isoflavones or phytoestrogen were excluded. The reason for this was related to their different impacts on body weight compared with soy (Akhlaghi et al., 2017[1]). Further reasons for exclusion were lack of data regarding means and SDs or SEs or IQR or 95 % CI of metabolic parameters, including both healthy subjects and MetS cases in the study.

### Data extraction 

The following data were extracted from the relevant articles: the first author's last name, year of publication, number of participants, age, sex, study design, type of intervention and control groups, study duration, MetS definition and mean and standard deviation of the metabolic parameters. When different soy-based foods were assessed in a single study in comparison with a control group, each intervention was regarded as a separate trial. In studies which provided repeated measures in different follow-up duration, the longest duration was included.

### Statistical analysis

The differences between the baseline and end values of metabolic parameters were calculated and considered as the mean change in any intervention arm. In studies which reported median and IQR, we assumed median equals to mean and estimated SD through dividing IQR by 1.35 (Hozo et al., 2005[15]). The pooled effect of the intervention was estimated by using weighted mean difference (WMD) and their corresponding 95 % CIs. The statistical heterogeneity between studies was examined by I^2 ^statistic, and values > 50 % were regarded as significant heterogeneity. When there was no evidence of heterogeneity between studies, pooled effect size was calculated using fixed-effect model; otherwise, random effects model was utilized. Subgroup analyses were performed to explore potential source of heterogeneity based on the country of study (Asia vs. non-Asia), soy types (soy protein vs. soy nut), design (parallel vs. cross-over), duration (< or > 10 wk), and substation of meat with soy (yes vs. no). Publication bias was evaluated by Begg's test and Egger's test and P < 0.05 was regarded as significant level. Sensitivity analysis was performed to evaluate the effect of any single effect size on the overall estimates. All analyses were done using Stata, version 11.2 (Stata Corp., College Station, TX, USA). P-values < 0.05 were considered statistically significant.

## Results

### Study characteristics

**Figure 1:**
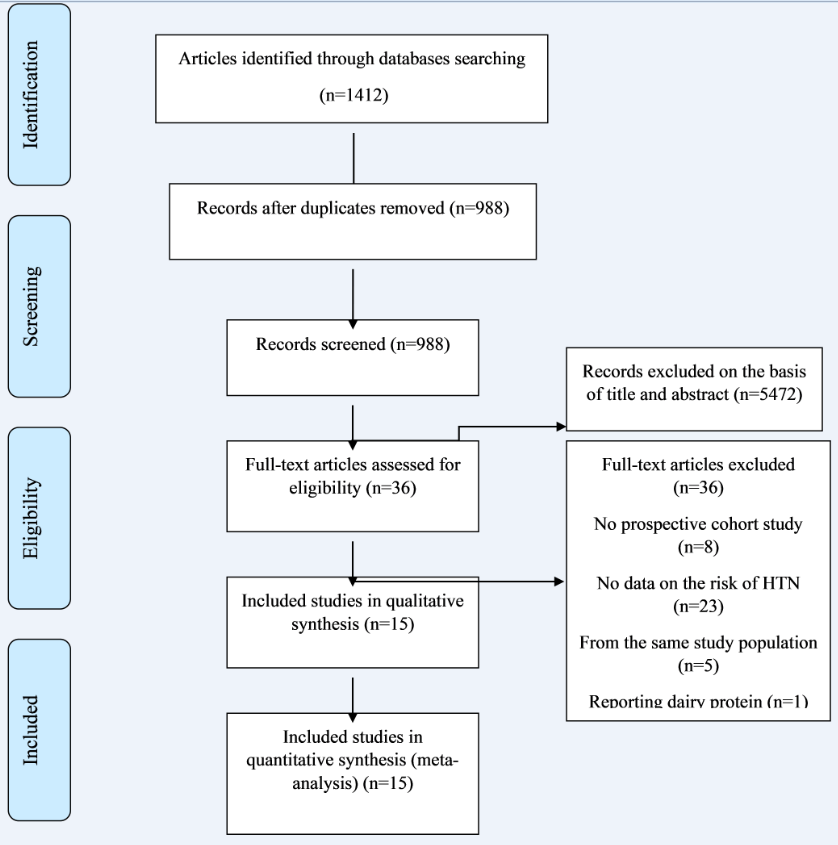
Flow chart of study selection

Our initial search resulted in 1412 articles, of which 424 were duplicate. After screening of 988 articles based on titles and abstracts, 952 articles were excluded and remaining 36 articles were assessed by full-text (Figure 1(Fig. 1)). Of these, six studies were identified which met our inclusion criteria for meta-analysis (Bakhtiari et al., 2012[8][6], 2019[7]; Ruscica et al., 2018[40]; Simao et al., 2012[43], 2014[44]; Winarsi et al., 2012[50]; Azadbakht et al., 2007[4]). In addition, we identified two more articles which either were non-randomized or reported percent change rather than baseline and end values of metabolic parameters; and therefore, they were only included in our systematic review (De Gregorio et al., 2017[10]; Bahls et al., 2011[5]) (Table 1(Tab. 1); References in Table 1: Azadbakht et al., 2007[4]; Bahls et al., 2011[5]; Bakhtiari et al., 2012[8][6], 2019[7]; De Gregorio et al., 2013[10]; Ruscica et al., 2018[40]; Simao et al., 2012[43], 2014[44]; Winarsi et al. 2012[50]). Five publications were derived from two studies (Bakhtiari et al., 2012[8], 2019[7]; Simao et al., 2012[43], 2014[44]; Winarsi et al., 2012[50]).

Two studies (four publications) examined the effect of soy nut and soy protein compared with no intervention (Bakhtiari et al., 2012[8][6], 2019[7]; Azadbakht et al., 2007[4]), one study (two publications) examined the effect of kinako and kinako plus fish oil with no intervention and fish oil (Simao et al., 2012[43], 2014[44]), which were included as separate effect sizes. Other four studies prescribed milk enriched with soy germ protein, soy protein, genistein, and kinako (Ruscica et al., 2018[40]; Winarsi et al., 2012[50]; De Gregorio et al., 2017[10]; Bahls et al., 2011[5]). The soy intake was between 25 and 35 g/d, and treatment duration ranged from 8 to 12 weeks. A total of 255 participants were included in the current meta-analysis.

### Serum lipids

**Figure 2:**
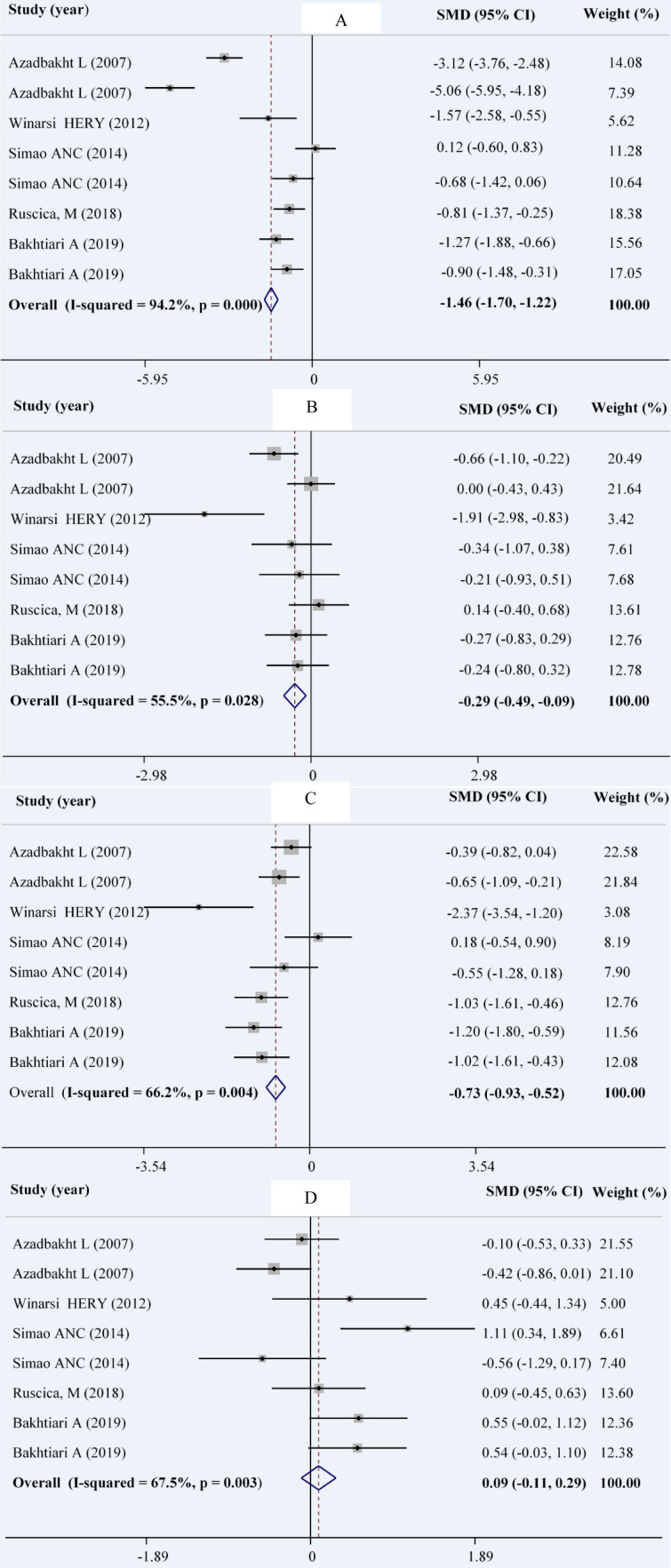
Forest plot showing the overall effect of soy consumption on total cholesterol (A), triglyceride (B), LDL-cholesterol (C) and HDL-cholesterol (D)

Combining a total of 9 comparisons from 5 studies (Ruscica et al., 2018[40]; Simao et al., 2014[44]; Winarsi et al., 2012[50]; Bakhtiary et al., 2012[8]; Azadbakht et al., 2007[4]) among 210 patients in intervention group in comparison with 211 patients in control group indicated a significant reduction in serum concentrations of TG (WMD: -0.29; 95 % CI: -0.49, -0.09 mg/dL; I^2^= 55.5 %), TC (WMD: -1.46; 95 % CI: -1.70, -1.22 mg/dL; I^2^= 94.2 %), and LDL-C (WMD: -0.73; 95 % CI: -0.93, -0.52 mg/dL; I^2^= 66.2 %). The same comparisons showed no significant change in serum levels of HDL-C in the intervention group compared with the control group (WMD: 0.09; 95 % CI: -0.11, 0.29 mg/dL; I^2^= 67.5 %) (Figures 2A-D(Fig. 2); References in Figure 2: Azadbakht et al., 2007[4]; Bakhtiari et al., 2019[7]; Ruscica et al., 2018[40]; Simao et al., 2014[44]; Winarsi et al. 2012[50]).

In our subgroup analysis, the effect of soy consumption on serum TG levels disappeared in non-Asian countries, in studies that lasted longer than 10 weeks, in studies that replaced red meat with soy products and in studies that investigated soy nuts. We also observed a significant increase in serum HDL-c in parallel studies (Table 2(Tab. 2)).

### Glycemic parameters

**Figure 3:**
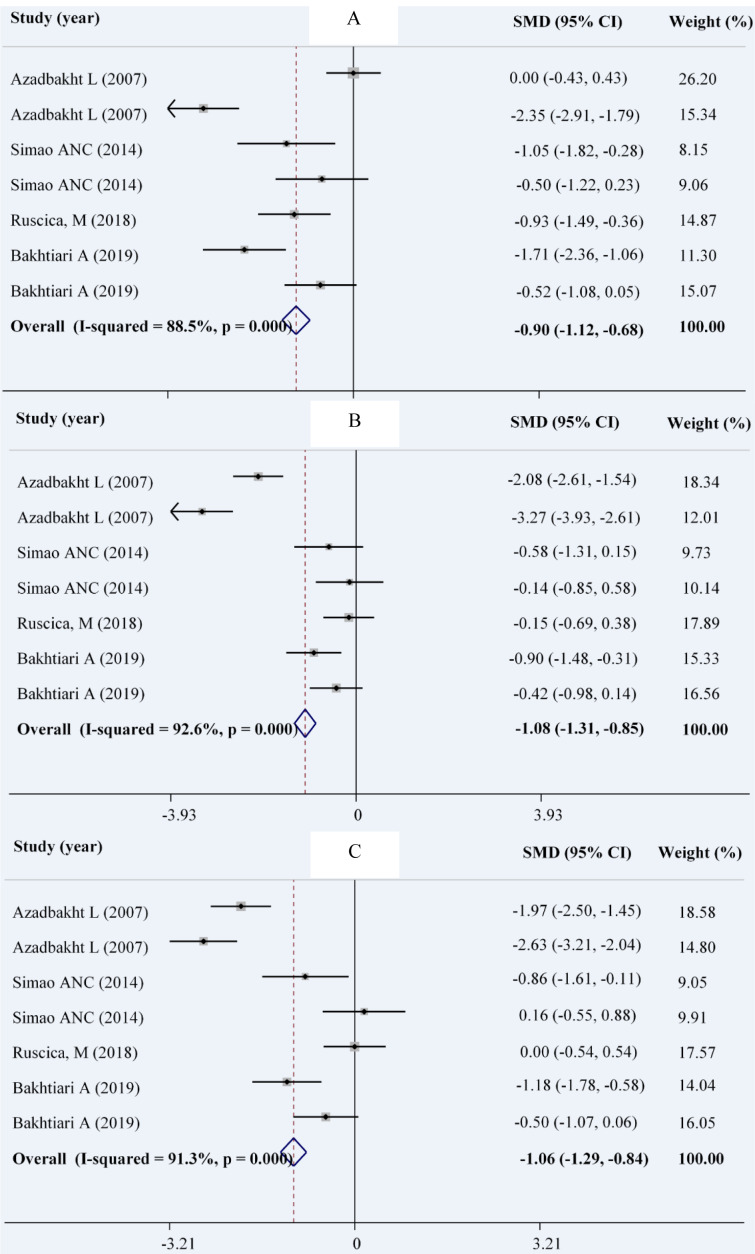
Forest plot showing the overall effect of soy consumption on fasting blood sugar (A), insulin (B) and HOMA (C)

Combining a total of 190 patients in the intervention group compared with 191 patients in the control group, derived from 4 RCTs with 7 effect sizes (Bakhtiari et al., 2019[7]; Simao et al., 2014[44]; Azadbakht et al., 2007[4]) indicated that soy intake could significantly reduce serum levels of FBS (WMD: -0.90; 95 % CI: -1.12, -0.68 mg/dL; I^2^= 88.5 %), insulin (WMD: -1.06; 95 % CI: -1.29, -0.84 pmol/ L; I^2^= 92.6 %) and HOMA-IR (WMD: -1.08; 95 % CI: -1.31, -0.85; I^2^= 91.3 %) (Figures 3A-C(Fig. 3); References in Figure 3: Azadbakht et al., 2007[4]; Bakhtiari et al., 2019[7]; Ruscica et al., 201[40]8; Simao et al., 2014[44]). The results of the subgroup analysis indicated no effect of soy consumption on serum insulin levels and HOMA-IR in non-Asian countries (Table 3(Tab. 3)).

### Anthropometric measures

**Figure 4:**
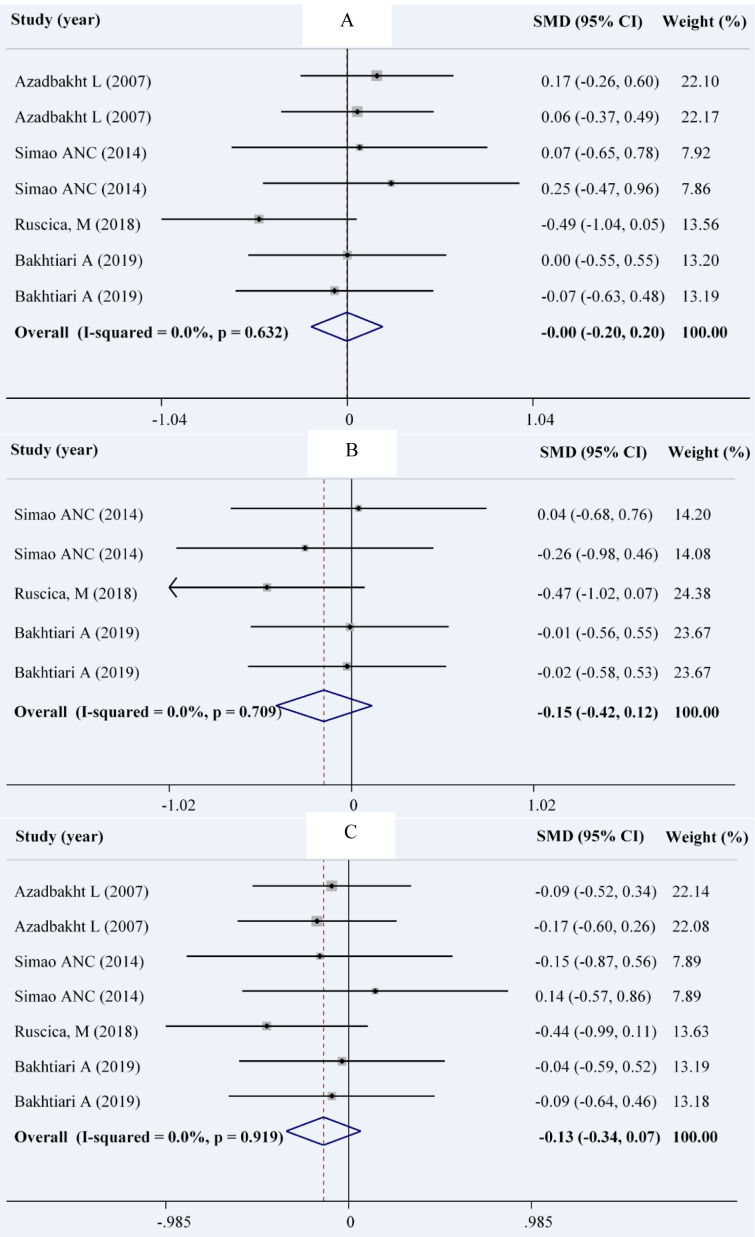
Forest plot showing the overall effect of soy consumption on weight (A), body mass index (B) and waist circumference (C)

According to our analysis on 4 studies with 7 effect sizes in 210 patients in the intervention group and 211 patients in the control group (Ruscica et al., 2018[40]; Simao et al., 2014[44]; Bakhtiari et al., 2012[8]; Azadbakht et al., 2007[4]), we found no significant change in weight (WMD: -0.00; 95 % CI: -0.20, 01.20 kg; I^2^= 0.0 %) and WC (WMD: -0.13; 95 % CI: -0.34, 0.07 cm; I^2^= 0.0 %). A total of 5 comparisons derived from 3 studies (Ruscica et al., 2018[40]; Simao et al., 2014[44]; Bakhtiari et al., 2012[8]) among 106 patients in the intervention group and 107 patients in the control groups showed no significant decrease in BMI (WMD: -0.15; 95 % CI: -0.42, 0.12 mg/dL; I^2^= 0.0 %) (Figures 4A-C(Fig. 4); References in Figure 4: Azadbakht et al., 2007[4]; Bakhtiari et al., 2019[7]; Ruscica et al., 2018[40]; Simao et al., 2014[44]).

### Blood pressure

**Figure 5:**
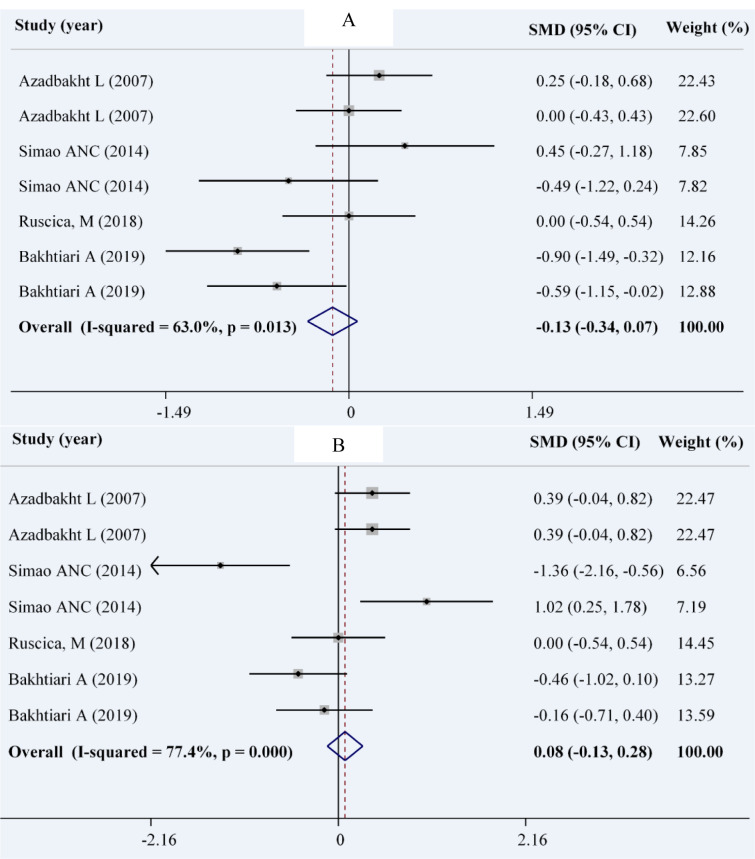
Forest plot showing the overall effect of soy consumption on systolic blood pressure (A) and diastolic blood pressure (DBP) (B)

We included 4 studies with 7 effect sizes to assess the effect of soy on systolic and diastolic blood pressure (Bakhtiari et al., 2019[7]; Ruscica et al., 2018[40]; Simao et al., 2012[43]; Azadbakht et al., 2007[4]). There were 190 patients in the soy group and 191 patients in the control group. Using the random effects model, there was no significant reduction in SBP (WMD: -0.13; 95 % CI: -0.34, 0.07 mmHg; I^2^= 63 %, P= 0.013) in the intervention group compared with the control group. Similarly, no significant change was observed in DBP (WMD: 0.08; 95 % CI: -0.13, 0.28 mmHg; I^2^= 77.4 %, P< 0.001) (Figures 5A and 5B(Fig. 5); References in Figure 5: Azadbakht et al., 2007[4]; Bakhtiari et al., 2019[7]; Ruscica et al., 2018[40]; Simao et al., 2014[44]). 

The subgroup analysis revealed a significant decrement in SBP in parallel studies, studies that lasted for more than 10 weeks, and studies that did not replace red meat with soy products. However, in cross-over trials, longer studies and those which replaced red meat with soy products, a significant increase was found in DBP (Table 4(Tab. 4)).

### Sensitivity analysis and publication bias 

The sensitivity analysis revealed that the removal of no specific studies significantly changed the effect of soy intake on BPs, serum lipids, glycemic status or anthropometric characteristics. There was no evidence of publication bias for studies examining the effect of soy consumption on SBP (Begg's test, P= 0.37; Egger's test, P= 0.45), DBP (Begg's test, P= 0.18; Egger's test, P= 0.34), TC (Begg's test, P= 0.27; Egger's test, P= 0.36), TG (Begg's test, P= 0.17; Egger's test, P= 0.20), LDL-C (Begg's test, P= 0.39; Egger's test, P= 0.27), HDL-C (Begg's test, P= 0.17; Egger's test, P= 0.19), FBS (Begg's test, P= 0.55; Egger's test, P= 0.36), insulin (Begg's test, P= 0.88; Egger's test, P = 0.99), HOMA-IR (Begg's test, P= 0.88; Egger's test, P= 0.57), weight (Begg's test, P= 0.88; Egger's test, P= 0.66), BMI (Begg's test, P= 0.81; Egger's test, P= 0.77) and WC (Begg's test, P= 0.76; Egger's test, P= 0.61).

## Discussion

Although to date many systematic reviews and meta-analyses have evaluated the effects of soy on metabolic parameters, to the best of our knowledge, this is the first one summarizing its effect in patients with MetS. 

The findings of the current meta-analysis demonstrated that soy products decreased glycemic markers (FBS, serum insulin levels, and HOMA-IR) and improved serum lipids (TG, TC, and LDL-C) in patients with MetS independent of any change in BMI or WC. No significant change was found in HDL-C, systolic and diastolic blood pressures following soy consumption. The subgroup analysis indicated that results for BMI, WC, FBS, TC, and LDL-C were not influenced by any confounders; however, other metabolic parameters were affected by geographical region or replacing meat with soy or soy type or study design and duration. 

In line with our findings, numerous meta-analyses have confirmed beneficial effects of soy on cardiovascular risk factors. In a recent umbrella review of systematic reviews and meta-analyses of observational studies and clinical trials, an inverse association was found between soy intake and serum levels of total cholesterol, LDL-C, and TG (Hemati et al., 2020[14]). The exact mechanisms underpinning reductions in serum cholesterol levels following soy intake are not clear (Lovati et al., 2000[21]). It is probable that peptides derived from soy protein digestion regulate cholesterol homeostasis in hepatic cells (Lovati et al., 2000[21]). Indeed, soy protein decreases serum LDL-C through both intrinsic and extrinsic (displacement) mechanisms, and it is one of few foods that reduces serum cholesterol either when added to the diet or when exchanged for saturated fatty acids and cholesterol (Jenkins et al., 2010[16]). Moreover, even though soy products are a rich source of isoflavones, it seems that their beneficial effects on serum cholesterol are not related to their isoflavones (Lovati et al., 2000[21]), but to other nutrients such as poly unsaturated fatty acids, fiber, stanols and sterols, and micronutrients content (Sacks et al., 2006[41]; Liu et al., 2014[20]).

Despite a consensus regarding the effects of soy on serum TC and LDL, debates remain in terms of the effects of soy on HDL-C, anthropometric measures and blood pressure. Our results, in consistent with some meta-analyses (Moradi et al., 2020[27]; Anderson et al., 1995[3]), revealed no significant change in serum HDL-C. However, Simao et al. reported that higher soy intake led to a mean increase of 1.1 mg/dL in serum HDL-C concentrations (Simao et al., 2014[44]). Similar inconsistencies have also been found for blood pressure. Whilst higher soy isoflavones intake lowered systolic and diastolic blood pressures in hypertensive individuals in a meta-analysis, it had no significant effect on normotensive subjects (Liu et al., 2012[18]). Our results are compatible with the earlier meta-analysis because mean SBP was less than 140 mmHg (as the cut-off point for hypertension) in all included studies in our meta-analysis. However, when we categorized studies according to the cut-off point for elevated blood pressure (130 mmHg), we observed that soy intake could effectively reduce SBP only in studies in which mean SBP was less than 130 mmHg. Given that soy can improve blood pressure through various mechanisms like improving flow mediated dilation (FMD) (Li et al., 2010[17]), inhibiting inflammatory processes (Nasca et al., 2008[30]), and stimulating NO production (Mahn et al., 2005[23]), difference in these factors may influence the results. Therefore, future studies are warranted to clearly explore the association between soy and blood pressure as well as the potential mechanisms underlying these associations.

We identified no association between soy and BMI and WC. These associations were also independent of geographical region, substitution for meat, and types of soy products. Consistently, earlier meta-analyses demonstrated a null association between soy products and obesity-related anthropometric measures (Akhlaghi et al., 2017[1]; Mu et al., 2019[28]); nevertheless, they showed that this association may vary by the geographical region and resulted in a significant reduction in body weight, BMI and WC in Asians but not Westerners, and with stronger effects in non-menopausal women (Mu et al., 2019[28]) or even led to an increase in body weight in short-term interventions (1-3 months), in obese individuals and in studies which supplemented with > 40 g/d soy protein (Akhlaghi et al., 2017[1]). In comparison with these meta-analyses, all included studies in our meta-analysis were conducted among overweight or obese subjects, supplemented with < 40 g/d soy products for less than 3 months, and all except for one enrolled postmenopausal women or men. It is worth mentioning that functions of enzymes and pathways involved in fatty acids metabolism are different between premenopausal and postmenopausal women (Misso et al., 2005[25]; Edwards et al., 2013[12]). Therefore, it is probable that non-significant associations found in our meta-analysis are, at least partly, explained by the menopause status of participants, and further RCTs are required in this context. 

Soy products consumption had favorable effects on FBS, HOMA, insulin, and serum TG concentrations. After subgroup analysis based on geographical region, the associations for HOMA, insulin, and TG remained significant only in Asians but not in Westerners. The beneficial effects of soy products on glycemic parameters might be attributed to soy-derived isoflavones and genistein (Glisic et al., 2018[13]). Isoflavones are known as selective estrogen receptor modulator since they prefer binding to and stimulating estrogen receptor beta rather than estrogen receptor alpha (Oseni et al., 2008[32]). Some studies indicated that phytoestrogens play an important role in the glucose and lipid metabolism through regulating peroxisome proliferator activator receptor (PPAR)-regulated genes, the sterol regulatory element binding protein (SREBP) (Mullen et al., 2004[29]; Ronis et al., 2009[38]), downregulation of genes involved in gluconeogenesis (Quinn and Yeagley, 2005[33]), upregulation of glucose homeostasis genes (Talaei and Pan, 2015[47]), and even at cellular levels in pancreatic islet cells, hepatocytes and intestinal cells (Vedavanam et al., 1999[49]; Mackowiak et al., 1999[22]; Szkudelska et al., 2000[46]). The difference between Asians and Westerners might be related to higher prevalence of S-equol producers among Asians compared with Westerners (Sekikawa et al., 2019[42]). S-equol, a metabolite of daidzein produced by gut microbiota, leads to the most antiatherogenic effects among isoflavones (Zhang et al., 2012[53]). 

Our meta-analysis has several strengths. First, our search strategy included various types of soy products which made this research more instructive; nevertheless, to date, only soy protein and nut have been investigated in this regard. Second, we performed several subgroup analyses to explore the source of heterogeneity. Third, our results were robust according to the sensitivity analysis and there was no evidence of publication bias between the studies. However, the limitations of the present meta-analysis must be kept in mind when interpreting the results. Our meta-analysis included only a few RCTs with small sample size and short follow-up duration. Furthermore, all studies, but not one (Ruscica et al., 2018[40]), were conducted only among women, particularly postmenopausal women that may differently respond to soy supplementation. Finally, studies included in our meta-analysis predominantly examined the intrinsic effects of soy products rather than the extrinsic effects. It is likely that replacement of meat with soy products (the extrinsic effects) are more helpful in comparison with adding soy products to the usual diet. 

In summary, this meta-analysis suggests that supplementation with soy products in patients with MetS is a helpful dietary intervention to reduce TG, TC, LDL-C, FBS, serum insulin, and HOMA-IR. Although most of the studies followed participants for 12 weeks, beneficial effects of soy consumption were also observed in studies that lasted for eight weeks. These effects were independent of BMI and WC change. Due to the paucity of studies in this regard, further studies with longer duration examining various types of soy products in both men and women are expected to explore the effects of soy products' supplementation in patients with MetS.

## Acknowledgement

The authors alone are responsible for the content and writing of the paper. 

## Funding

This study was funded by Isfahan University of Medical Sciences.

## Conflict of interest

The authors declare that there are no conflicts of interest.

## Authors’ contribution

FH and FS contributed to conception, search and data extraction. FH and NM contributed to analysis and manuscript drafting. All authors contributed to review of the manuscript and approved the final draft for submission.

## Figures and Tables

**Table 1:**
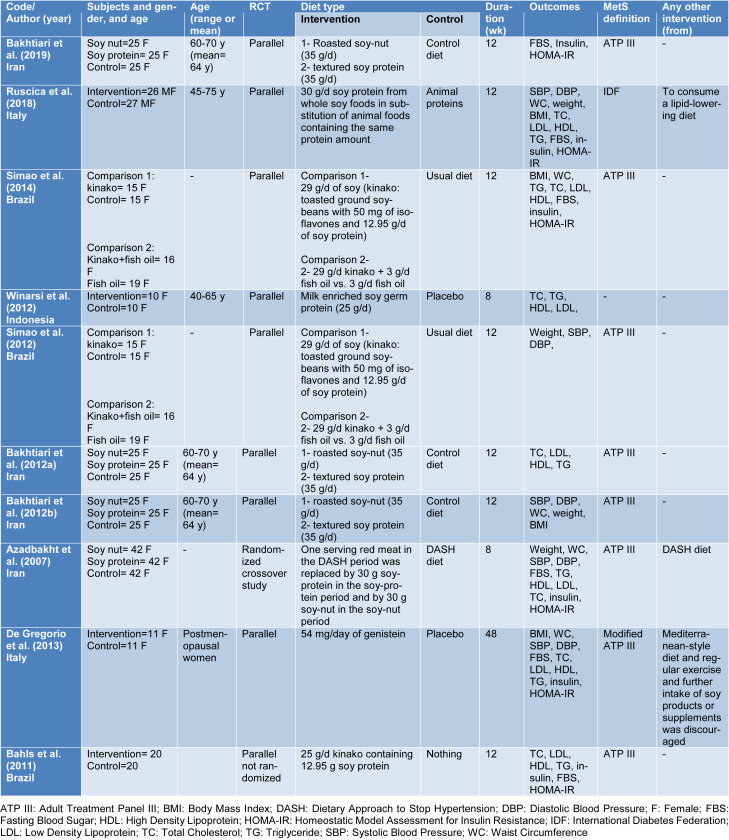
Characteristics of studies included in this meta-analysis

**Table 2:**
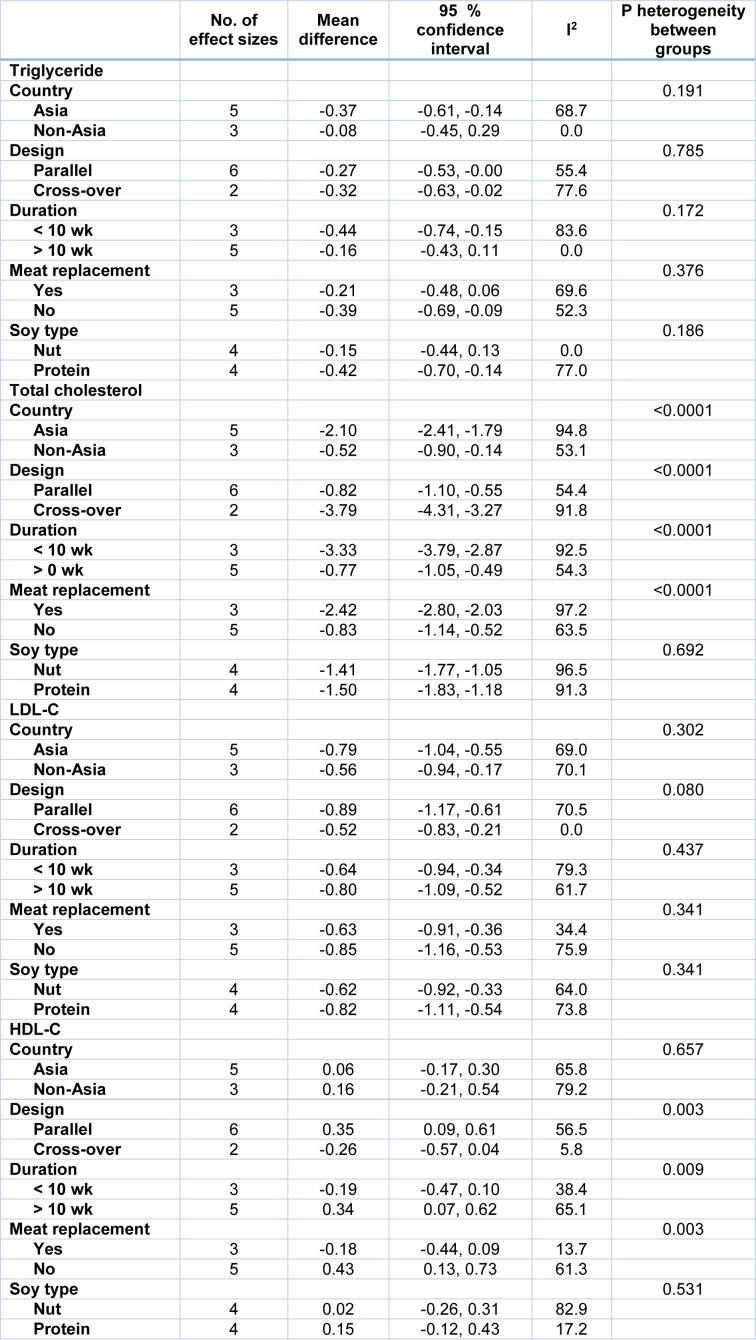
Subgroup analysis for the effect of soy on serum lipids in patients with metabolic syndrome

**Table 3:**
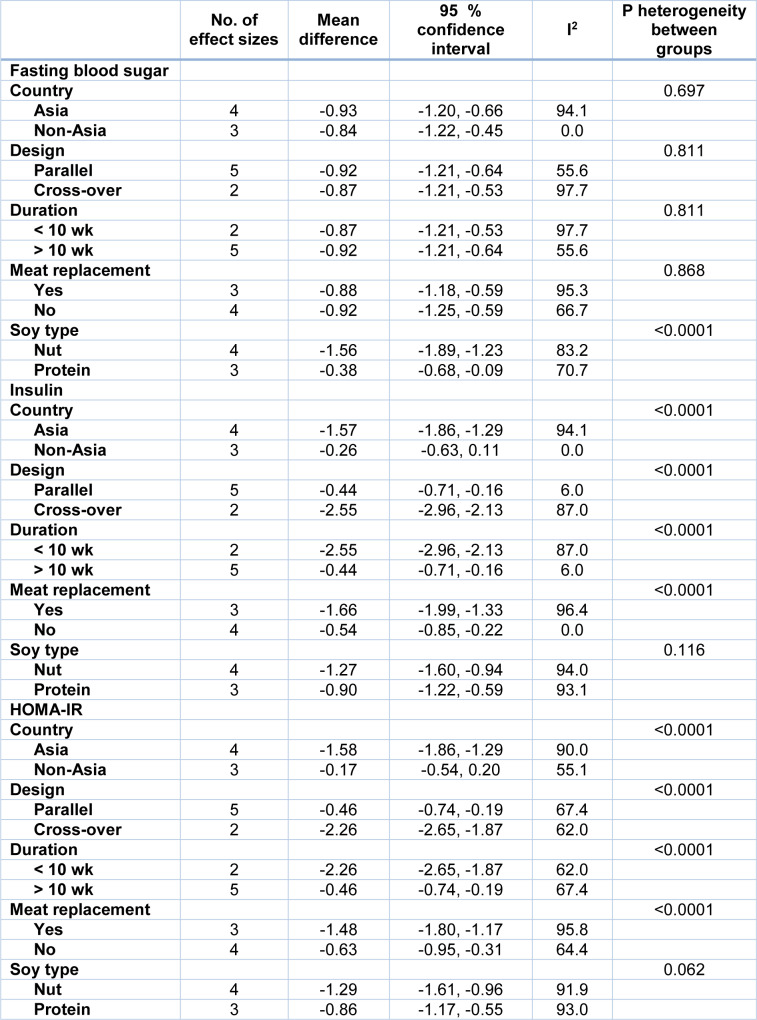
Subgroup analysis for the effect of soy on glycemic parameters in patients with metabolic syndrome

**Table 4:**
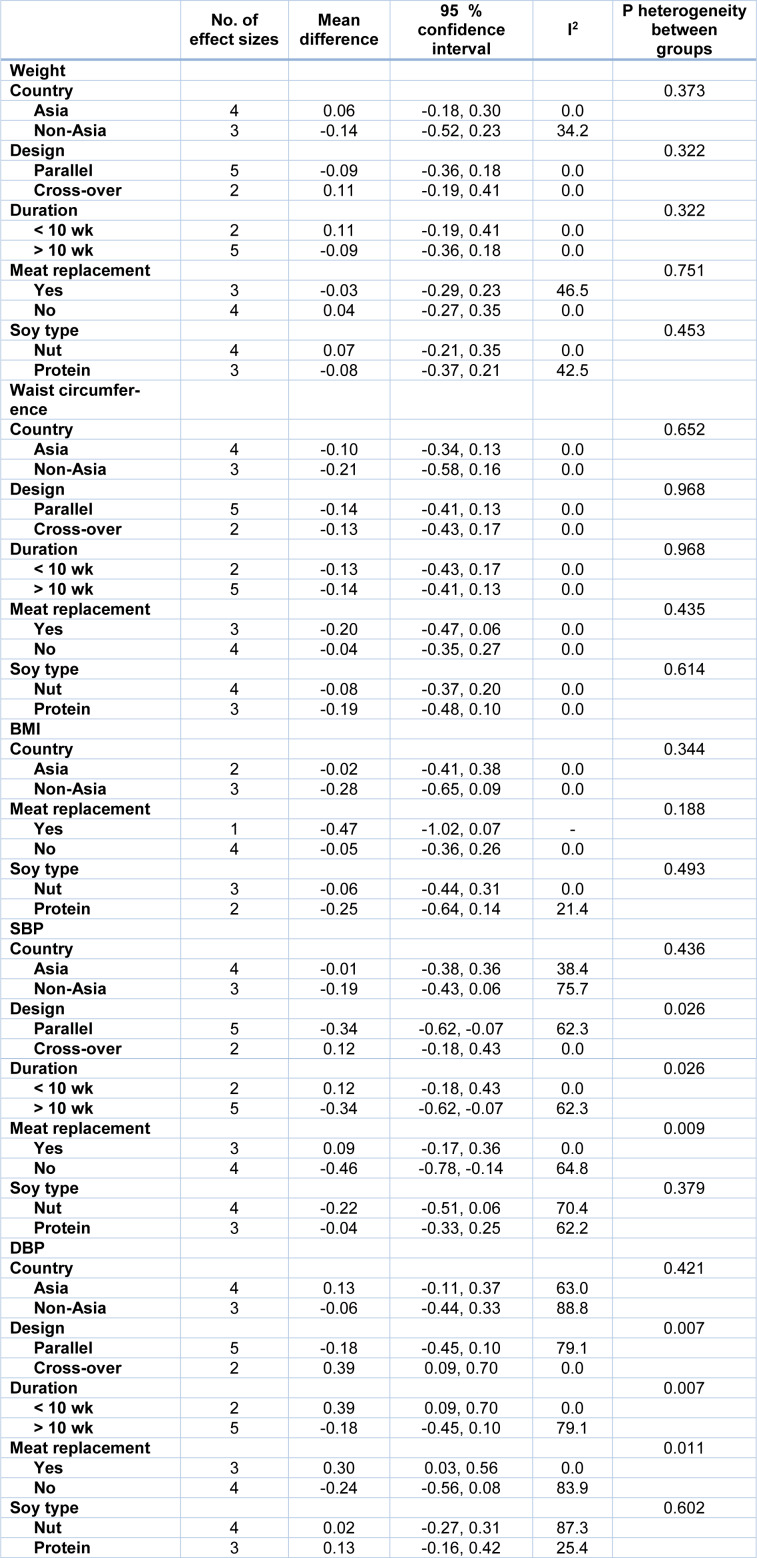
Subgroup analysis for the effect of soy on anthropometric measures and blood pressure in patients with metabolic syndrome

## References

[1] Akhlaghi M, Zare M, Nouripour F (2017). Effect of soy and soy isoflavones on obesity-related anthropometric measures: A systematic review and meta-analysis of randomized controlled clinical trials. Adv Nutr.

[2] Alissa EM, Ferns GA (2012). Functional foods and nutraceuticals in the primary prevention of cardiovascular diseases. J Nutr Metab.

[3] Anderson JW, Johnstone BM, Cook-Newell ME (1995). Meta-analysis of the effects of soy protein intake on serum lipids. N Engl J Med.

[4] Azadbakht L, Kimiagar M, Mehrabi Y, Esmaillzadeh A, Padyab M, Hu FB (2007). Soy inclusion in the diet improves features of the metabolic syndrome: a randomized crossover study in postmenopausal women. Am J Clin Nutr.

[5] Bahls LD, Venturini D, Scripes ND, Lozovoy MAB, Simao TNC, Simao ANC (2011). Evaluation of the intake of a low daily amount of soybeans in oxidative stress, lipid and inflammatory profile, and insulin resistance in patients with metabolic syndrome. Arq Bras Endocrinol Metabol.

[6] Bakhtiar[y]i A, Yassin Z, Hanachi P, Rahmat A, Ahmad Z, Jalali F (2012). Effects of soy on metabolic biomarkers of cardiovascular disease in elderly women with metabolic syndrome. Arch Iran Med.

[7] Bakhtiari A, Hajian-Tilaki K, Omidvar S, Nasiri-Amiri F (2019). Clinical and metabolic response to soy administration in older women with metabolic syndrome: a randomized controlled trial. Diabetol Metab Syndr.

[8] Bakhtiari A, Yassin Z, Hanachi P, Rahmat A, Ahmad Z, Sajadi P (2012). Effects of soy on body composition: a 12-week randomized controlled trial among iranian elderly women with metabolic syndrome. Iran J Public Health.

[9] Cassidy A, Brown JE, Hawdon A, Faughnan MS, King LJ, Millward J (2006). Factors affecting the bioavailability of soy isoflavones in humans after ingestion of physiologically relevant levels from different soy foods. J Nutr.

[10] De Gregorio C, Marini H, Alibrandi A, Di Benedetto A, Bitto A, Adamo EB (2017). Genistein supplementation and cardiac function in postmenopausal women with metabolic syndrome: Results from a pilot strain-echo study. Nutrients.

[11] de la Iglesia R, Loria-Kohen V, Zulet MA, Martinez JA, Reglero G, Ramirez de Molina A (2016). Dietary strategies implicated in the prevention and treatment of metabolic syndrome. Int J Mol Sci.

[12] Edwards MH, Gregson CL, Patel HP, Jameson KA, Harvey NC, Sayer AA (2013). Muscle size, strength, and physical performance and their associations with bone structure in the Hertfordshire Cohort Study. J Bone Miner Res.

[13] Glisic M, Kastrati N, Musa J, Milic J, Asllanaj E, Portilla Fernandez E (2018). Phytoestrogen supplementation and body composition in postmenopausal women: A systematic review and meta-analysis of randomized controlled trials. Maturitas.

[14] Hemati N, Asis M, Moradi S, Mollica A, Stefanucci A, Nikfar S (2020). Effects of genistein on blood pressure: A systematic review and meta-analysis. Food Res Int.

[15] Hozo SP, Djulbegovic B, Hozo I (2005). Estimating the mean and variance from the median, range, and the size of a sample. BMC Med Res Methodol.

[16] Jenkins DJ, Mirrahimi A, Srichaikul K, Berryman CE, Wang L, Carleton A (2010). Soy protein reduces serum cholesterol by both intrinsic and food displacement mechanisms. J Nutr.

[17] Li S-H, Liu X-X, Bai Y-Y, Wang X-J, Sun K, Chen J-Z (2010). Effect of oral isoflavone supplementation on vascular endothelial function in postmenopausal women: A meta-analysis of randomized placebo-controlled trials. Am J Clin Nutr.

[18] Liu X, Li S, Chen J, Sun K, Wang X, Wang X (2012). Effect of soy isoflavones on blood pressure: a meta-analysis of randomized controlled trials. Nutr Metab Cardiovasc Dis.

[19] Liu ZM, Chen YM, Ho SC (2011). Effects of soy intake on glycemic control: A meta-analysis of randomized controlled trials. Am J Clin Nutr.

[20] Liu ZM, Ho SC, Chen YM, Ho S, To K, Tomlinson B (2014). Whole soy, but not purified daidzein, had a favorable effect on improvement of cardiovascular risks: A 6-month randomized, double-blind, and placebo-controlled trial in equol-producing postmenopausal women. Mol Nutr Food Res.

[21] Lovati MR, Manzoni C, Gianazza E, Arnoldi A, Kurowska E, Carroll KK (2000). Soy protein peptides regulate cholesterol homeostasis in Hep G2 cells. J Nutr.

[22] Mackowiak P, Nogowski L, Nowak K (1999). Effect of isoflavone genistein on insulin receptors in perfused liver of ovariectomized rats. J Recept Signal Transduct Res.

[23] Mahn K, Borrás C, Knock GA, Taylor P, Khan IY, Sugden D (2005). Dietary soy isoflavone induced increases in antioxidant and eNOS gene expression lead to improved endothelial function and reduced blood pressure in vivo. FASEB J.

[24] Mazidi M, Rezaie P, Ferns GA, Gao HK (2016). Impact of different types of tree nut, peanut, and soy nut consumption on serum C-reactive protein (CRP): A systematic review and meta-analysis of randomized controlled clinical trials. Medicine.

[25] Misso ML, Jang C, Adams J, Tran J, Murata Y, Bell R (2005). Differential expression of factors involved in fat metabolism with age and the menopause transition. Maturitas.

[26] Moher D, Liberati A, Tetzlaff J, Altman DG (2009). Preferred reporting items for systematic reviews and meta-analyses: The PRISMA statement. Ann Intern Med.

[27] Moradi M, Daneshzad E, Azadbakht L (2020). The effects of isolated soy protein, isolated soy isoflavones and soy protein containing isoflavones on serum lipids in postmenopausal women: A systematic review and meta-analysis. Crit Rev Food Sci Nutr.

[28] Mu Y, Kou T, Wei B, Lu X, Liu J, Tian H (2019). Soy products ameliorate obesity-related anthropometric indicators in overweight or obese asian and non-menopausal women: A meta-analysis of randomized controlled trials. Nutrients.

[29] Mullen E, Brown RM, Osborne TF, Shay NF (2004). Soy isoflavones affect sterol regulatory element binding proteins (SREBPs) and SREBP-regulated genes in HepG2 cells. J Nutr.

[30] Nasca MM, Zhou J-R, Welty FK (2008). Effect of soy nuts on adhesion molecules and markers of inflammation in hypertensive and normotensive postmenopausal women. Am J Cardiol.

[31] O'Neill S, O'Driscoll L (2015). Metabolic syndrome: A closer look at the growing epidemic and its associated pathologies. Obes Rev.

[32] Oseni T, Patel R, Pyle J, Jordan VC (2008). Selective estrogen receptor modulators and phytoestrogens. Planta Med.

[33] Quinn P, Yeagley D (2005). Insulin regulation of PEPCK gene expression: a model for rapid and reversible modulation. Curr Drug Targets Immune Endocr Metabol Disord.

[34] Reaven GM (1995). Pathophysiology of insulin resistance in human disease. Physiol Rev.

[35] Rios DR, Rodrigues ET, Cardoso AP, Montes MB, Franceschini SA, Toloi MR (2008). Effects of isoflavones on the coagulation and fibrinolytic system of postmenopausal women. Nutrition.

[36] Rizzo G, Baroni L (2018). Soy, soy foods and their role in vegetarian diets. Nutrients.

[37] Rochlani Y, Pothineni NV, Kovelamudi S, Mehta JL (2017). Metabolic syndrome: Pathophysiology, management, and modulation by natural compounds. Adv Cardiovasc Dis.

[38] Ronis MJ, Chen Y, Badeaux J, Badger TM (2009). Dietary soy protein isolate attenuates metabolic syndrome in rats via effects on PPAR, LXR, and SREBP signaling. J Nutr.

[39] Ruscica M, Gomaraschi M, Mombelli G, Macchi C, Bosisio R, Pazzucconi F (2014). Nutraceutical approach to moderate cardiometabolic risk: Results of a randomized, double-blind and crossover study with Armolipid Plus. J Clin Lipidol.

[40] Ruscica M, Pavanello C, Gandini S, Gomaraschi M, Vitali C, Macchi C (2018). Effect of soy on metabolic syndrome and cardiovascular risk factors: A randomized controlled trial. Eur J Nutr.

[41] Sacks FM, Lichtenstein A, Van Horn L, Harris W, Kris-Etherton P, Winston M (2006). Soy protein, isoflavones, and cardiovascular health: An American Heart Association Science Advisory for professionals from the Nutrition Committee. Circulation.

[42] Sekikawa A, Ihara M, Lopez O, Kakuta C, Lopresti B, Higashiyama A (2019). Effect of S-equol and soy isoflavones on heart and brain. Curr Cardiol Rev.

[43] Simao ANC, Lozovoy MAB, Bahls LD, Morimoto HK, Simao TN, Matsuo T (2012). Blood pressure decrease with ingestion of a soya product (kinako) or fish oil in women with the metabolic syndrome: role of adiponectin and nitric oxide. Br J Nutr.

[44] Simao ANC, Lozovoy MAB, Dichi I (2014). Effect of soy product kinako and fish oil on serum lipids and glucose metabolism in women with metabolic syndrome. Nutrition.

[45] Stanaway JD, Afshin A, Gakidou E, Lim SS, Abate D, Abate KH (2018). Global, regional, and national comparative risk assessment of 84 behavioural, environmental and occupational, and metabolic risks or clusters of risks for 195 countries and territories, 1990–2017: A systematic analysis for the Global Burden of Disease Study 2017. Lancet.

[46] Szkudelska K, Nogowski L, Szkudelski T (2000). Genistein affects lipogenesis and lipolysis in isolated rat adipocytes. J Steroid Biochem Mol Biol.

[47] Talaei M, Pan A (2015). Role of phytoestrogens in prevention and management of type 2 diabetes. World J Diabetes.

[48] Tokede OA, Onabanjo TA, Yansane A, Gaziano JM, Djousse L (2015). Soya products and serum lipids: a meta-analysis of randomised controlled trials. Br J Nutr.

[49] Vedavanam K, Srijayanta S, O’Reilly J, Raman A, Wiseman H (1999). Antioxidant action and potential antidiabetic properties of an isoflavonoid‐containing soyabean phytochemical extract (SPE). Phytother Res.

[50] Winarsi H, Wijayanti SPM, Purwanto A (2012). Soy germ protein with or without-Zn improve plasma lipid profile in metabolic syndrome women. HAYATI J Biosci.

[51] Xu H, Li X, Adams H, Kubena K, Guo S (2018). Etiology of metabolic syndrome and dietary intervention. Int J Mol Sci.

[52] Yang G, Shu XO, Jin F, Zhang X, Li HL, Li Q (2005). Longitudinal study of soy food intake and blood pressure among middle-aged and elderly Chinese women. Am J Clin Nutr.

[53] Zhang X, Gao Y-T, Yang G, Li H, Cai Q, Xiang Y-B (2012). Urinary isoflavonoids and risk of coronary heart disease. Int J Epidemiol.

[54] Zhang XM, Zhang YB, Chi MH (2016). Soy protein supplementation reduces clinical indices in type 2 diabetes and metabolic syndrome. Yonsei Med J.

